# Supplementary material for: A machine learning framework for the evaluation of myocardial rotation in patients with noncompaction cardiomyopathy
Source: PLoS One. 2021 Nov 29;16(11):e0260195. doi: 10.1371/journal.pone.0260195 (PMC8629285; doi:10.1371/journal.pone.0260195)
Supplement: Attachment — Submitted filename: Response to reviewrs.docx [file pone.0260195.s001.docx]

To: Zhifan Gao

Academic Editor PLOS ONE Journal

Dear Dr. Gao

We are thrilled by the opportunity to resubmit our revised manuscript. On behalf of all co-authors, I would like to thank you and the invited reviewers for the time and effort dedicated to reviewing our work, as well as for the revisions that we believe have strengthened this paper.

We have made every effort to address all points and issues raised. Enclosed please see detailed response to each of the reviewers’ comments to the manuscript. We believe that the information provided in this manuscript has significant clinical value and should be communicated to the scientific community through this journal.

The funders had no role in study design, data collection and analysis, decision to publish, or preparation of the manuscript.

The authors received no specific funding for this work and the authors have declared that no competing interests exist.

Sincerely,

Vera Maria Cury Salemi, MD, PhD

Corresponding author

**Answers to Reviwers**

**Reviewer #1:** In this study, authors presented a study of NCC identification by using ML method. They focused on evaluation of the RBR criteria. They stated that good performance was obtained. However, there were several significant issues that the result was therefore not convincing.

Comments:

1. The language usage throughout this paper need to be improved, the author should do some proofreading on it. Give the article a mild language revision to get rid of few complex sentences that hinder readability and eradicate typo erros.

Thank you for the comments. We have made all the efforts to correct them.

2. Overall, the basic background is not introduced well. To my understanding, differentiation between NCC patient to non NCC patient was the key objective. However, no explanation about the ‘3 most common criteria’ was provided while limited information about the RBR without explanation about the correlation between RBR and NCC and the correlation between RBR and other echocardiographic parameters. It was confusing that whether the NCC or the RBR was the objective of this work.

In this study, a random forest algorithm was used to find the best echocardiographic predictors to rigid body rotation (RBR) pattern in noncompaction cardiomyopathy (NCC) patients, which was consistent with worse systolic, diastolic, and myocardium deformation indices, parameters usually important for the risk stratification of different cardiomyopathies.

First of all, it´s important to say that our primary objective was not to validate RBR as a diagnostic parameter in NCC patients. Although there are no gold standard diagnostic criteria for this entity, all patients enrolled in this study were regularly attended in the heart failure department, and matched the “3 most common” echocardiographic (Chin, Jenni Stollberguer) and cardiac magnetic resonance criteria, which improves the accuracy of this diagnosis in our sample.

Although the endocardial hypertrabeculation involvement in NCC patients may affect left ventricular myocardial torsion, it remains unknown if patients with impaired myocardial deformation will subsequently develop deterioration of global function or if myocardial dysfunction will remain stable. New prognostic parameters are necessary to better stratify these patients. As a hypothesis-driven study, we explored the potential role of RBR pattern in the risk stratification in NCC patients, and our results should be interpreted in the context of an exploratory analysis.

3. Introduction needs to explain the main contributions of the work more clearly. The novelty of this paper is not apparent.

Aiming to better explain the potential role of RBR pattern in the risk stratification of NCC patients, we amended the introduction section as follows:

*“Although the role of myocardial strain analysis in risk stratification of patients with LV hypertrabeculation remain unknow, we hypothesize that RBR may have a potential value in the risk stratification of patients who fulfilled the clinical and imaging criteria of NCC. In that context, we developed and tested ML framework, consisting of a random forest algorithm and a neural network, to evaluate the best echocardiographic predictors to RBR pattern in NCC patients, providing additional information for risk stratification of these patients, without the necessity of image post processing in dedicated workstations.”*

4. Literature review techniques have to be strengthened. The authors should consider more recent research done in the field of their study.

Additional references were included as suggested. We would like to highlight in the introduction section:

*“In the current era of precision medicine, machine learning techniques has been related to many potential applications in cardiac imaging. Nevertheless, there are few recent papers that explored artificial intelligence tools in NCC patients, above all focused on refining the current diagnostic criteria (REF 1)or on the prediction of adverse clinical outcomes (REF 2) using data extracted from echocardiography and cardiac magnetic resonance, without correlations with myocardial strain analysis”*

1. Bricq S, Frandon J, Bernard M, Guye M, Finas M, Marcadet L, Miquerol L, Kober F, Habib G, Fagret D, Jacquier A, Lalande A. Semiautomatic detection of myocardial contours in order to investigate normal values of the left ventricular trabeculated mass using MRI. J Magn Reson Imaging. 2016 Jun;43(6):1398-406.
2. Rocon C, Tabassian M, Tavares de Melo MD, de Araujo Filho JA, Grupi CJ, Parga Filho JR, Bocchi EA, D'hooge J, Salemi VMC. Biventricular imaging markers to predict outcomes in non-compaction cardiomyopathy: a machine learning study. ESC Heart Fail. 2020 Oct;7(5):2431-2439.

5. The method section is not clear. Above all, it is not clear that what kinds of parameters were collected. How did you collected the parameters that were not mentioned in method section. What is the characteristic of the data. Second, it is unclear whether the image quality check was only performed on data for LS analysis? What about the other parameters?

The method section was significantly changed according these questions. Please check the updated version of the manuscript. Of note, clinical baseline parameters are described in Table 1, and all echocardiographic parameters are displaced in table 2.

6. There is a missing of citation for the random forest algorithm. How did you determine the key features? It is not clear about the architecture of the MLP. Also, it is not clear about the split of data for training, validation and test.

Thank you for catching up this. The missing citation is now included (reference 16).

[16] Menze, B. H., Kelm, B. M., Masuch, R., Himmelreich, U., Bachert, P., Petrich, W., & Hamprecht, F. A. A comparison of random forest and its Gini importance with standard chemometric methods for the feature selection and classification of spectral data. *BMC bioinformatics*, *10*(1), 1-16. (2009)

Regarding the selection of key features, the features were selected using a correlation matrix (Figure 3) and then organized in decreasing order according to the influence on the Random Forest classification (Figure 4). Only one additional feature (LV GLS) was included in the model due to its clinical relevance.

Regarding the architecture of the MLP, Figure 2 illustrates the neural network architecture used, including the organization of layers and neurons (input layer 11, hidden layers: 256, 256, and 128, output layer: 2).

To answer the other questions, we amended “Multilayer perceptron neural networks” subsection, as follows:

*“*The Scikit-learn library was used to provide the implementation of this neural network. A k-fold validation approach with k = 5 was used to validate the model, corresponding to a training dataset with 60% of the samples, testing with 20%, and validation with 20%. The training stages of 3000 times each, and the learning rate is equal to 0.001.*”*

7. Result section. Correlation was presented, but where was the result of RF for parameters selection?

Sorry if it was not clear, but the result of RF for parameters selection was displaced in Figure 4.

8. It is unclear how NC/C was generated.

Thank you. We inserted a sentence regarding this point in the methods section:

*“Jenni criterion was used to calculate the N/C ratio, which measures the maximal end-systolic ratio of non-compacted to compacted layers.”*

9. Discussion section. what is boundary subsets?

This is a mathematical term and was removed in order to avoid misinterpretations.

10. Authors stated that ‘cardiac mechanics seems to be more promising as a specific marker, and trabeculae may be considered just a red flag cardiac mechanics.' However, while the cardiac mechanics' parameters, ie. GLS and LVEF were the results of various phenotypes, it is not clear how the authors came to this statement. Please elaborate.

Thank you for your comments. We agree that this a very controversial question, beyond the scope of this study. We removed the referred statement.

REVIEWER 2:

This paper uses a machine learning (ML) based model to evaluate myocardium function, and the role of echocardiographic predictors for rigid body rotation in Noncompaction cardiomyopathy (NCC). The manuscript is clear, straightforward, easy to follow. The results are sufficient and convincing. I think this study has the potential for NCC risk stratification. However, I would like to draw the author's attention to the following major concerns:

1)In this study, the authors used 50 patients for training and testing the machine learning model. Considering, the model is a deep neural network. The data set the size of 50 patients is insufficient.

Due to the small dataset available, the model was exposed to some problems, such as underfitting, which causes high rates of errors. In order to overcome this limitation, the k-fold validation method was applied. In this approach, the same data is divided into k-subsets in which all have the same size. So, the model was carried out k-time, using a k-subset as a test dataset, another k-subset as a validation dataset. Finally, the remaining k-subset was used as a training dataset. In this way, each subset was reused during executions such as training, testing, and validation. In the end, the result is obtained from the average of the metrics of the executions.

2)The authors do not explain clearly its used deep neural network. As a key point, what is the architecture of the deep neural network? Does the network use a general architecture, or is it specially optimized for NCC? Do different network architectures change the results？The authors should do a more thorough literature survey. Just to name a few:

-Cardiac Functional Analysis with Cine MRI via Deep Learning Reconstruction

-Segmentation and quantification of infarction without contrast agents via spatiotemporal generative adversarial learning.

-Automated left ventricular dimension assessment using artificial intelligence developed and validated by a UK-wide collaborative

Thank you for your suggestion. We appreciated the suggested articles and we have added one of them in the introduction section. Other new related references were included.

The architecture of the deep neural network is illustrated in figure 2, including the organization of layers and neurons (input layer: 11, hidden layers: 256, 256, and 128, output layer: 2).

Dataset size is a challenge limitation that makes it difficult to use a complex network architecture since such networks demand large volumes of data. For this reason, we chose to use an MLP network with few layers organized in a sequential structure and with an approach directed to the types of numerical data present in the problem. There is no singularity in noncompaction patients’ data. We only selected a clinical problem and applied the MLP in order to figure out which echocardiography parameters would be predictors of RBR.

3) In this study, the authors used a single echocardiographic ultrasound system from a single center. I think this point is the most important limitation of this study. The generalization error is a big problem for machine learning (ML) based models. It would be better to discuss this point in the manuscript.

We agree with your point. Intervendors machine variability is a serious concern, mainly in strain analysis. We add this sentence in the limitation session:

*“*Third, all analysis was performed in only one software, we could not test the algorithm with different vendors which reduce its applicability. While there is a joint effort among the vendors to diminish these discrepancies*”*

Reference: Farsalinos KE, Daraban AM, Ünlü S, Thomas JD, Badano LP, Voigt JU. Head-to-Head Comparison of Global Longitudinal Strain Measurements among Nine Different Vendors: The EACVI/ASE Inter-Vendor Comparison Study. J Am Soc Echocardiogr. 2015 Oct;28(10):1171-1181, e2.

4) There are some grammar errors and typos. I suggest the authors make an solid, overall proofreading.

Thank you for the comments. We have made all the efforts to correct them.
